# Reply to Comment on Ultrasound Guidance for Botulinum Neurotoxin Chemodenervation Procedures. *Toxins* 2018, *10*, 18—Quintessential Use of Ultrasound Guidance for Botulinum Toxin Injections

**DOI:** 10.3390/toxins10100400

**Published:** 2018-09-28

**Authors:** Katharine E. Alter, Barbara I. Karp

**Affiliations:** 1 Functional and Applied Biomechanics Section, Rehabilitation Medicine, Clinical Center, National Institutes of Health, Bethesda, MD 20892-1604, USA; 2 Combined Neurosciences IRB, National Institutes of Health, Bethesda, MD 20892-1604, USA; karpb@ninds.nih.gov

We thank the authors for their detailed letter and salient comments related to our article on Ultrasound Guidance for botulinum toxin (BoNT) injections.

We and the authors of the letter agree that ultrasound (US) guidance is the most anatomically accurate localization method for BoNT injections. We also agree that the outcomes and or safety of BoNT procedures may be enhanced by using US guidance as well as by combining the use of US with other localization methods such as electromyography, motor endplate and/or innervation targeting zone targeting.

In their first comment, the authors advocate for the use of the term “innervation zone targeting” rather than “motor endplate targeting”. They then eloquently review the topic of motor end plates and motor innervation zones as it related to BoNT injections and suggest that motor endplate and motor innervation zone targeting should be considered separate techniques. We note that the terminology used in the literature for these regions varies, with some authors using the term “motor endplates” or “motor endplate zones” while other authors utilize the term “innervation zone” [1,2,3,4,5]. We use such commonly accepted terminology in our paper; the particular issue raised of more specific terminology remains outside of the scope of this paper. To enhance communication between clinicians and researchers perhaps, as the authors of the letter suggest, this terminology should be standardized to reflect the differences between motor endplates and motor innervation zones. We urge that the authors of the letter publish further on this topic so as to promote the use of a consistent terminology among clinicians and researchers.

With respect to US guidance for nerve blocks, the authors state that we denied that US is useful for selective motor nerve imaging. They appear to have misunderstood our statement. In fact, we stated that **motor endplates** cannot be visualized using current US imaging technology while clearly stating that US, especially when combined with electrical stimulation (E-Stim), is useful for anesthetic and/or neurolytic nerve or motor point blocks. Our practice is to use US combined with E-Stim for every nerve block procedure. US is used to identify the safest path to the target nerve as well as to position the needle next to, but not within, the nerve. We have also found that using US for nerve block procedures also decreases “on time” of the stimulator when it is delivering current, thereby decreasing stimulation-related discomfort during the procedure. However, such a detailed discussion of US guidance for sensory or motor nerve blocks or motor point block is outside the scope of this chapter, which focuses on US guidance for botulinum toxin (BoNT) injections.

In their second comment, the letter states “concerning particular muscles, we strongly disagree with the authors regarding tibialis posterior, iliopsoas, and sternocleidomastoid injections”. We are unclear about the exact nature of their disagreement. The authors of the letter then go on to comment on the location of innervation zones within these muscles and how to target injections. Their point of disagreement may relate to a misinterpretation of what we were aiming to demonstrate with our Figure 5a,d and Figure 6b. The purpose of these figures (and others) was to demonstrate the appearance of muscles when imaged in a transverse/short axis versus longitudinal/long axis scans. The images of the iliopsoas and tibialis posterior were included to demonstrate the advantage of transverse/short axis imaging for muscle identification using pattern recognition. These figures were not included to advocate for or against a specific approach or site of needle insertion into these muscles. Detailed information about the approach or site of needle insertion for specific muscles is also beyond the scope of our mandate, which was to review the *technique* of US for BoNT injections. We refer the readers to several articles recently published by the letter’s authors, which cover this topic in detail [6,7,8,9].

In their next comment, the authors of the letter advocate for the use of an “in-plane” needle insertion technique. Regarding in-plane (IP) and out-of-plane (OP) procedural guidance techniques, both the letter and our article point out the potential limitations of an OP approach. In our article, we state that “One technique may be superior to the other for a given patient or particular muscle/structure. Therefore, clinicians should be familiar with both approaches.” We stand by this statement. While the OP technique has its limitations, it also has potential advantages in certain situations and, when performed correctly utilizing a walk-down technique, can be safe and effective for accurate needle placement [10]. As stated in our article, specific situations where the OP technique may be preferable to an IP approach include when

- The IP technique requires insertion of the needle distant to the target such that the needle would traverse un-targeted structures.
- There is no safe path to the target using an IP technique.
- An OP approach provides direct access to the muscle, such as the flexor carpi radialis, while the IP technique would require the needle to penetrate or traverse additional undesired muscles or pass near to or through other structures such as vessels or nerves.

In the end, the sonographer/injector must select the injection method based on a number of factors including optimal target location/visualization, structures in the path of the needle, and/or the injector’s assessment of the safest technique for the procedure being performed.

Lastly, the authors comment on the topic of toxin dose and dilution, including high volume versus low volume injections. There is limited literature that informs optimal BoNT product concentration and volume of injection for particular muscle targets and conditions. In general, more dilute concentrations/higher volume injections are recommended by the manufacturers of BoNT products when treating spasticity to enhance spread of toxin within relatively large muscles. In contrast, high concentration/small volume injections are required to accurately deliver very low doses of toxin, such as those required for blepharospasm. Higher concentration/small volume injections are often recommended in small muscles and in patients with focal dystonia [5,11]. As with the issue of IP or OP approach to a muscle, the dose and dilution must be selected by the injector based on a number of clinical considerations in addition to and including targeting of innervation zones. We agree with the authors that additional studies comparing dilution, dose, and location of injection within a muscle/target will be helpful in resolving the ongoing questions about optimizing benefit and minimizing side effects when using intramuscular botulinum toxin.

## Figures and Tables

**Figure 5:**
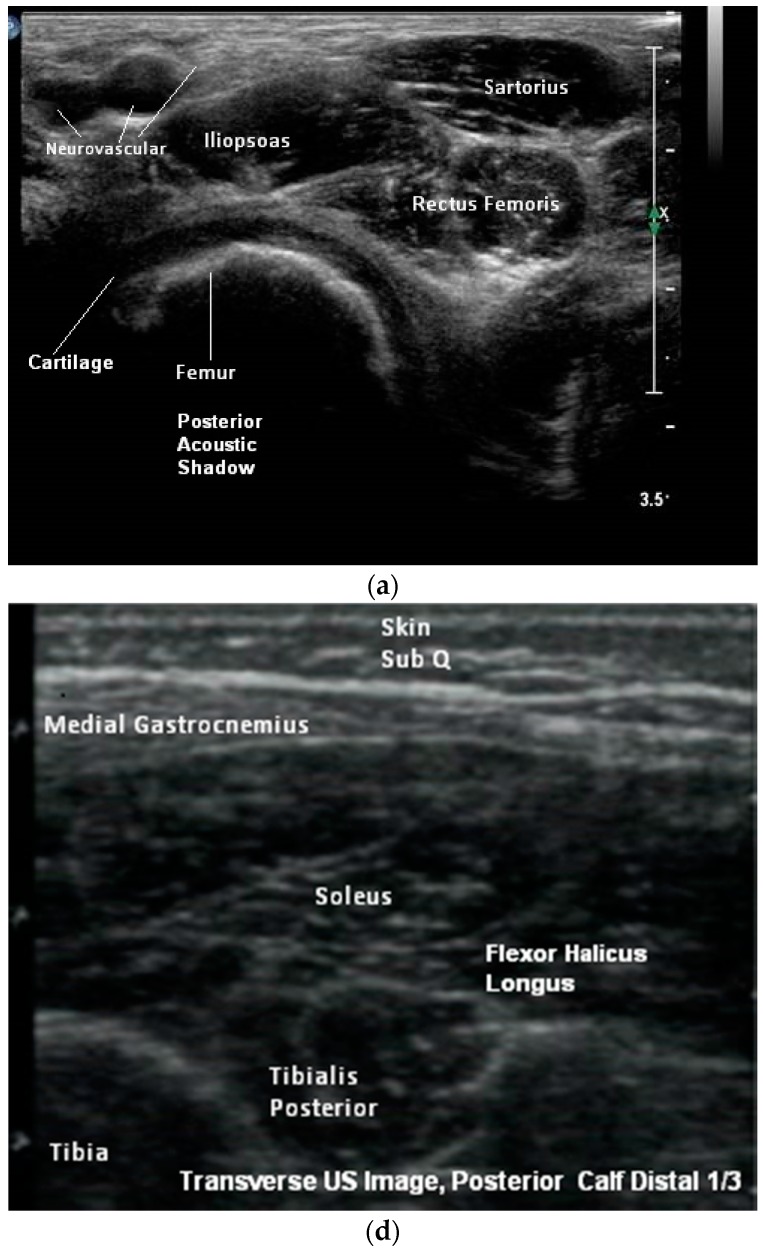
(**a**) Transverse B-mode ultrasound (US) Image, Proximal Thigh; (**d**) Transverse B-mode US Image, Posterior Calf (Distal 1/3).

**Figure 6:**
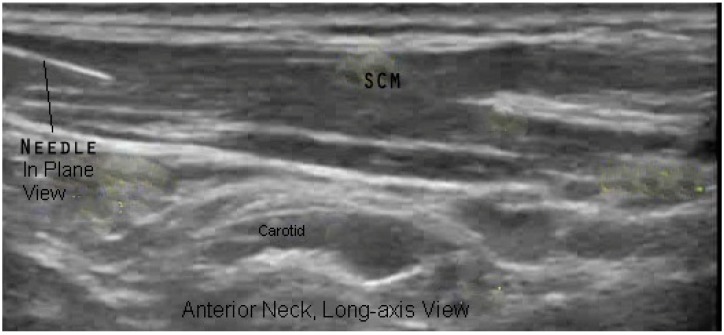
(**b**) Long-axis B-Mode US Image, Anterior neck.
